# The Power of Environment: A Comprehensive Review of the Exposome’s Role in Healthy Aging, Longevity, and Preventive Medicine—Lessons from Blue Zones and Cilento

**DOI:** 10.3390/nu17040722

**Published:** 2025-02-18

**Authors:** Silvana Mirella Aliberti, Mario Capunzo

**Affiliations:** 1 Department of Medicine, Surgery and Dentistry “Scuola Medica Salernitana”, University of Salerno, 84081 Salerno, Italy; mcapunzo@unisa.it; 2 Complex Operational Unit Health Hygiene, University Hospital “San Giovanni di Dio e Ruggi d’Aragona”, 84131 Salerno, Italy

**Keywords:** exposome, environment, aging, longevity, protective factors, risk factors, health and disease, Blue Zones, Cilento, preventive medicine

## Abstract

Aging and longevity are shaped by the exposome, a dynamic network of environmental, social, and biological factors. Understanding how these exposures interact with biological mechanisms can inform strategies for healthier aging. **Background/Objectives**: This review explores the exposome as a dynamic system encompassing both protective and risk factors, with a specific focus on how beneficial environmental exposures, microbiome diversity, lifestyle behaviors, and resilience mechanisms contribute to successful aging. By analyzing high-longevity populations, such as the Blue Zones and Cilento, it aims to identify common determinants of successful aging. **Methods**: A mixed-method study was conducted, combining a systematic review of the English literature (2003–2024) with a comparative analysis of longevity regions. A structured search was performed in PubMed, Scopus, and Google Scholar using keywords such as “longevity”, “Blue Zones”, “Cilento”, “microbiome”, “environmental factors”, and related terms. Additionally, qualitative and quantitative analysis were applied to assess key protective factors across different aging models. **Results**: This study identified key factors contributing to successful aging in longevity hotspots, including sustained exposure to biodiverse natural environments, adherence to Mediterranean or plant-based diet rich in polyphenols and probiotics, regular physical activity, strong social networks, and psychological resilience. A novel aspect of this review is the role of the gut microbiome as a mediator between environmental exposures and immune–metabolic health, influencing inflammation modulation and cellular aging. Despite geographic and cultural differences, case studies reveal a shared pattern of protective factors that collectively enhance lifespan and healthspan. **Conclusions**: The exposome is a critical determinant of aging trajectories, acting through complex interactions between environmental and biological mechanisms. By integrating insights from high-longevity populations, this mixed-method study proposes a comprehensive framework for optimizing microbiome health, enhancing resilience, and promoting protective environmental exposures. These findings provide a translational perspective to guide future interventions in aging research and global health initiatives.

## 1. Introduction

The journey of life is shaped not only by our genetic blueprint—the inherited set of genes that influences biological traits and predispositions—but also by the multitude of environmental influences we encounter throughout our lifetime. This cumulative environmental exposure is collectively known as the exposome, a framework that bridges genetics and environmental sciences, offering a holistic perspective on the determinants of healthy aging and longevity. Unraveling the intricate interplay between genetic and environmental factors is crucial for understanding aging trajectories and identifying strategies to promote health and longevity.

### The Interplay Between the Exposome and Healthy Aging

Aging is a multifactorial and dynamic process, influenced by genetic and environmental factors. The exposome, first introduced in 2005 [1], provides a comprehensive model for understanding how cumulative environmental exposures interact with biological systems to influence health outcomes. While genetic predispositions play a significant role in shaping biological traits, environmental factors significantly influence gene expression and disease susceptibility through mechanisms such as epigenetic modifications, oxidative stress, and inflammation [2]. These interactions emphasize the role of lifestyle choices and environmental management in longevity [3].

Macro- and micro-environmental factors play a crucial role in aging trajectories. Macro-environmental determinants, such as climate, land use, and water quality, establish the broader ecological conditions affecting population health [4,5]. In turn, micro-environmental factors, including diet, physical activity, socioeconomic status, and social relationships, directly shape biological aging [6,7]. Scientific evidence supports the importance of green spaces in urban environments, showing significant benefits for mental well-being, cardiovascular health, and overall longevity [8]. These findings highlight the need for public health strategies that incorporate green spaces into urban planning.

One of the key biological mechanisms mediating the effects of the exposome on aging is epigenetics, which refers to modifications that alter gene expression without altering the DNA sequence [9]. Environmental factors such as diet, physical activity, exposure to pollutants, and chronic stress can induce epigenetic changes that influence disease risk and longevity. Studies have shown that positive environmental exposures—such as the Mediterranean diet and strong community networks—are associated with improved longevity, whereas chronic stress and pollutants accelerate aging and increase the risk of noncommunicable diseases (NCDs) [10,11,12,13].

The impact of differential environmental exposures is evident in the gender health paradox, where women often outlive men but have higher rates of chronic disease and disability, in contrast, men typically have shorter lifespans but fewer years of poor health [14,15]. These differences underscore the complexity of aging and the need for a comprehensive approach that considers both genetic and environmental influences.

Global aging is a significant challenge as the world’s population reaches 8 billion, including more than 800 million people aged 65 years and older [16]. While increased life expectancy is a positive achievement, it has also led to an increasing burden of chronic disease [17]. The World Health Organization (WHO) estimates that non-communicable diseases (NCDs) account for 74% of the global disease burden, with environmental and behavioral factors playing a central role in their development [18].

Given these challenges, the implementation of preventive strategies aimed at reducing harmful environmental exposures and promoting healthier lifestyles is imperative. Populations with exceptional longevity, such as those in Okinawa, Nicoya, Ikaria, Loma Linda, Sardinia, Martinique, and Cilento, demonstrate that lifestyle, community support, and environmental factors contribute significantly to extended healthspan.

Notably, in our previous study, the Cilento region was identified as a potential new Blue Zone, highlighting the pivotal role of environmental factors, Mediterranean dietary patterns, intergenerational social ties, and a balanced lifestyle in promoting longevity [7]. Building upon this foundation, the present study extends the investigation by incorporating the exposome framework to assess the cumulative impact of lifestyle and environmental exposures on longevity. Given the authors’ familiarity with the Cilento region, a structured approach was taken to ensure analytical objectivity.

This review explores the role of the exposome in aging and longevity through a mixed-methods approach that integrates qualitative and quantitative analyses, including a structured scoring system. Specifically, it examines the influence of macro- and micro-environmental exposures on the aging process and lifespan, aiming to: (1) Identify protective factors that promote healthier aging trajectories; (2) Provide actionable strategies for improving quality of life, enhancing resilience, and reducing health disparities; (3) Bridge the gap between theoretical insights and practical applications, with a particular focus on translating lessons from natural longevity models, such as the Blue Zones and Cilento, into effective public health recommendations. By synthesizing existing evidence, this review aims to contribute to the development of a comprehensive global framework for promoting healthier aging and advancing public health strategies.

## 2. Materials and Methods

### 2.1. Study Design and Rationale

This study employs a mixed-methods approach, integrating a narrative synthesis, a qualitative case study, and quantitative analysis to investigate the determinants of longevity. The research framework is based on the exposome model, which considers environmental, nutritional, physical activity, microbiome, and psychosocial factors as cumulative exposures shaping health outcomes over time.

A comparative analysis was conducted between Cilento and the recognized Blue Zones, exploring key protective factors linked to longevity. This builds upon prior research [7] that identified similarities between Cilento and other longevity hotspots through a comparative study. The present work extends this investigation by applying the exposome framework to assess the cumulative impact of lifestyle and environmental factors on longevity in Cilento, emphasizing unique interactions that may differentiate it from previously recognized Blue Zones.

### 2.2. Literature Search and Selection Criteria

The literature review followed PRISMA guidelines to ensure methodological rigor. Studies were identified through systematic searches in PubMed/Medline, Scopus, and Google Scholar using Boolean operators and MeSH terms related to “longevity”, “Blue Zones”, “Cilento”, “nutrition”, “physical activity”, “microbiome”, “psychosocial resilience”, “environmental factors”. The search was restricted to studies published between 2003 and 2024 to capture the most relevant scientific advancements.

**Figure 1:**
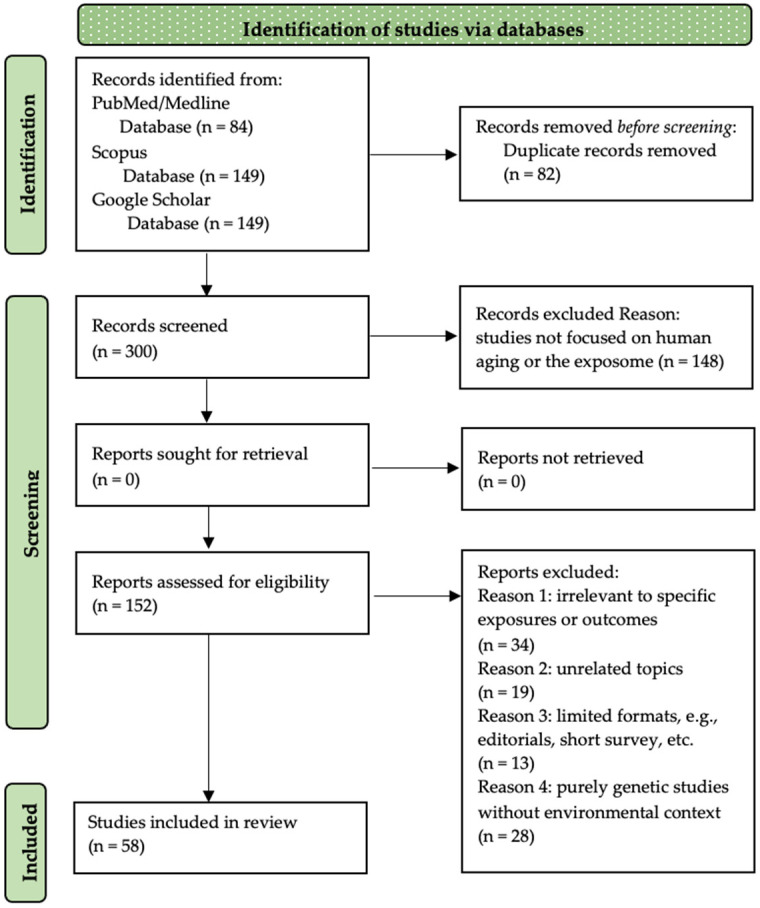
A flowchart illustrating the identification of studies for inclusion.

Selection Process:

○ Initial screening: Titles and abstracts were screened independently by two reviewers to ensure relevance.
○ Full-text review: The same reviewers assessed full texts based on predefined inclusion/exclusion criteria.
○ Resolution of discrepancies: Any disagreement was resolved through discussion with a third researcher to ensure reliability.
○ Flowchart: A PRISMA flow diagram (Figure 1) illustrates the selection process.

Inclusion criteria:

○ Studies assessing longevity determinants (e.g., environmental, nutrition, physical activity, psychosocial, and microbiome factors).
○ Studies providing quantitative or qualitative data on Blue Zones and Cilento.
○ Studies evaluating social determinants of health, including poverty, social class, and occupational factors as contributors to aging disparities.
○ Peer-reviewed research articles in English or Italian.

Exclusion criteria:

○ Studies focusing exclusively on genetic factors without environmental considerations.
○ Studies lacking methodological transparency or reliability.
○ Studies on populations outside the scope of longevity research.

### 2.3. Data Analysis and Case Study Methodology

Given the heterogeneity of data sources, a narrative synthesis was performed to integrate findings from different disciplines. The study used a mixed-methods case study approach to analyze longevity-related exposures in Cilento and compare them with the established Blue Zones.

Quantitative Analysis:

To assess demographic trends in longevity regions, we employed the aging tendency ratio (proportion of 65+ old population to the total population) rather than the centenarian ratio. This decision was driven by the challenge of obtaining consistent and annual centenarian data across all Blue Zones. For example, in Ikaria, the most recent comprehensive demographic records date back to 2011 census, making it difficult to track centenarian prevalence over time. The aging tendency ratio, while not a direct measure of centenarian prevalence, serves as a proxy for the overall aging structure of the population, providing insights into long-term demographic stability and aging trends.

○ The aging tendency ratio was estimated using demographic data.
○ Table 1 evaluates data availability and consistency in different longevity regions, addressing challenges in retrieving centenarian records in some Blue Zones.
○ Scores were assigned independently by two researchers based on predefined criteria, including data completeness, update frequency, and stratification by advanced age groups. Expert analyses and official demographic records were also considered. Final agreement on the scores was reached through consensus.

Qualitative Analysis:

○ Environmental, nutrition, physical activity, microbiome diversity, social support, and psychological resilience were analyzed through a thematic approach based on a systematic literature review.
○ A comparative evaluation was conducted to estimate the presence and impact of protective factors across different longevity regions. To quantify these influences, a scoring system (Table 2) was developed using a 5-point scale, where 1 indicates limited evidence or minimal impact, and 5 reflects strong evidence with a significant influence on longevity.
○ The scoring process was conducted by a panel of eight independent experts, including two researchers who are co-authors of this paper (SMA and MC), and six specialists: a local administrator, a geriatrician, a biogerontologist, a nutritionist, an environmental scientist, and a behavioral psychologist. Each expert independently evaluated the identified factors.
○ A modified Delphi methodology was adopted to refine the scoring process and achieve consensus. Following the initial individual assessments, results were shared among panel members, who were then allowed to adjust their scores based on group feedback. To ensure unbiased assessments and minimize external influences, anonymity and confidentiality of participants were maintained throughout the process. This approach encouraged open and independent evaluations, reducing the risk of dominant opinions affecting the consensus.
○ Reliability analysis was conducted using Kendall’s coefficient of concordance (W = 0.87, *p* < 0.001), confirming a high level of agreement among the panel members before reaching the final consensus.

The analysis focused on:

- The influence of macro- and micro-environmental exposures on aging.
- Biological mechanisms linking these exposures to aging outcomes.
- Evidence-based strategies for promoting healthy aging, particularly within population health interventions.

Reports from global health organizations (e.g., World Health Organization—WHO, Unit Nations—UN) were also reviewed to contextualize findings within broader demographic and public health trends.

The availability of annual demographic data is critical for maintaining the scientific validity and recognition of these regions as unique models of longevity. While environmental, lifestyle, social, dietary, and microbiome factors play a fundamental role, demographic consistency is also necessary to ensure that designed Blue Zones continue to meet longevity criteria over time. The lack of regularly updated centenarian data in certain regions highlights the need for systematic population records to support replicable and comparative studies. Establishing standardized demographic criteria would not only validate existing Blue Zones but also allow for the inclusion of new regions that exhibit similar longevity patterns.

The findings are structured to reflect a logical progression from theoretical foundations to practical applications:

○ Conceptual foundations—The exposome’s impact on aging is explored, highlighting key protective factors such as positive environmental exposures, nutrition, physical activity, social support, and the microbiome.
○ Risk factors in aging—The role of environmental stressors, pollution, and lifestyle-related risks in accelerating aging and disease susceptibility is examined.
○ Case studies of natural longevity models—A comparative analysis of the Blue Zones and Cilento illustrates how environmental and lifestyle factors interact to support healthy aging.

### 2.4. Author Positionality and Conflict of Interest Disclosure

The authors are based in Salerno, Italy, in close proximity to the Cilento region, and have extensive knowledge of its demographic, environmental, and cultural characteristics. This positionally provides valuable insight into the region’s longevity determinants while maintaining a rigorous and systematic approach to data analysis. The authors acknowledge this background and have taken measures to ensure objectivity, such as employing standardized evaluation criteria and independent verification of findings.

## 3. Protective Environmental and Lifestyle Factors in Aging

The aging process is characterized by a progressive decline in intrinsic biological functions, driven by mechanisms such as genomic instability, loss of proteostasis, telomere attrition, and epigenetic alterations [19,20]. While these mechanisms have a genetic basis, they are profoundly influenced by environmental and lifestyle factors, underscoring the central role of the exposome—the cumulative environmental exposures experienced from conception to death [4,6,21,22]. The exposome shapes aging trajectories by either accelerating biological dysfunctions or enhancing resilience and repair mechanisms.

**Figure 2:**
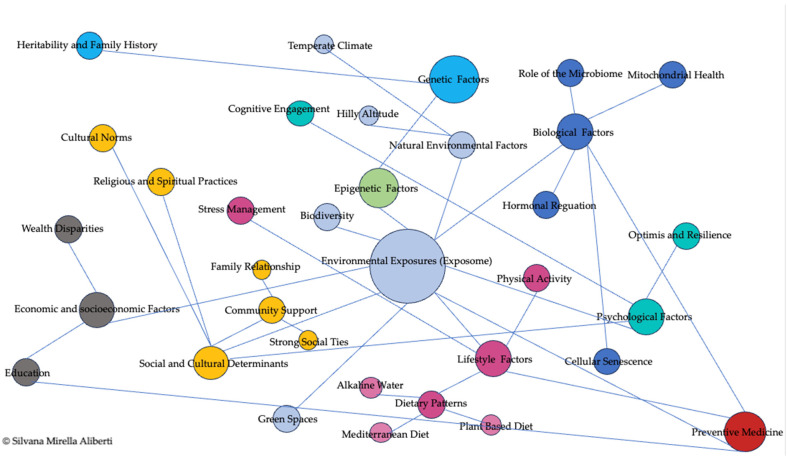
Conceptual diagram showing the interrelationships between Lifespan and Longevity Factors. Note: This conceptual diagram visualizes the interrelated factors influencing longevity, structured into primary domains, subcategories, and further subdivisions. The diagram was constructed using Microsoft PowerPoint, following a hierarchical classification of factors derived from the existing literature on aging and the exposome. Node size reflects the hierarchical level of each factor: larger nodes represent broad categories (e.g., Environmental Exposures), medium-sized nodes indicate subcategories (e.g., Lifestyle Factors, Biological Factors), and smaller nodes denote specific determinants within each subcategory (e.g., Dietary Pattern, Mitochondrial Health). Regarding the placement of Genetic Factors, while it could be embedded within Biological Factors, it has been represented as a separate domain to emphasize its distinct contributions to longevity, independent of modifiable environmental influences. Different colors are used to distinguish primary domains and their subcategories, visually highlighting their interrelationships. This color-coding enhances clarity and emphasizes the interdependencies across categories, illustrating how different determinants collectively shape aging trajectories. The number of connections was determined based on well-documented interactions in the literature, prioritizing relationships with the strongest empirical support. While additional links could be drawn, the visualization aims to balance comprehensiveness with clarity, preventing excessive complexity while maintaining the integrity of key interconnections.

To promote healthy aging at both individual and societal levels, a comprehensive framework must integrate multiple protective factors that sustain biological resilience and mitigate age-related decline. As illustrated in Figure 2, key contributors include favorable environmental exposures, balanced nutrition, regular physical activity, a diverse and robust microbiome, and strong social and psychological support networks. The following sections will explore these components, highlighting their interactions with biological mechanisms that influence longevity and well-being.

### 3.1. Natural Environments and Exposure to Green Spaces

A growing body of evidence highlights the critical role of natural environments in promoting health, resilience, and longevity [23,24,25,26,27]. While genetic factors account for only 15–40% of lifespan variability [28,29,30,31,32], environmental exposures—including access to green spaces—play a dominant role in shaping aging trajectories. Forests, parks, coastal areas, and mountainous regions, have been consistently associated with lower stress levels, improved cardiovascular function, and enhanced immune responses. Even passive exposure, such as viewing greenery, can reduce cortisol levels, improve mood, and support cognitive function [33,34,35,36].

The biophilia hypothesis suggests an innate human connection with nature, supported by research linking regular interaction with green spaces to reduced stress and lower risk of neurodegenerative diseases [37]. Similarly, the biodiversity hypothesis highlights the role of natural environments in shaping the human microbiome [38], enhancing immune balance, and reducing inflammation-related diseases [38,39,40]. Activities such as hiking promote microbial diversity, which is essential for immune resilience, whereas urbanization and limited natural exposure have been associated with increased chronic disease risk [41,42,43,44,45,46,47].

Climate and natural resources further modulate health and longevity. Mediterranean-like climates, with mild temperatures (17–20 °C), support outdoor activity and chronic disease prevention [4,7,48,49]. Additionally, access to mineral-rich water sources, as observed in longevity regions like Cilento and Hunza, has been linked to cardiovascular and metabolic health benefits [5,50,51].

Beyond physiological benefits, natural environments foster mental well-being and social engagement, reducing anxiety, depression, and cognitive decline risks [52,53,54]. Exposure to natural light also regulates circadian rhythms, improving sleep quality and cognitive function in aging populations [55,56].

In urban settings, integrating green infrastructure, such as parks, pedestrian-friendly streets, and community gardens, is essential for supporting the mobility and independence of older adults [57,58]. These spaces encourage physical activity while mitigating the risks of social isolation and cognitive impairment.

In summary, natural landscapes, clean water, balanced climates, and microbial diversity are integral to healthy aging. Public health strategies and urban planning should prioritize these factors to enhance longevity, resilience, and well-being across the generations (Figure 2).

### 3.2. Balanced Nutrition and Dietary Patterns

Nutrition plays a fundamental role in modulating aging mechanisms and promoting longevity [59,60] (Figure 2). Dietary patterns significantly influence oxidative stress, inflammation, and mitochondrial function—key processes involved in cellular senescence and the onset of chronic diseases. Diets rich in whole, nutrient-dense foods such as fruits, vegetables, whole grains, nuts, seeds, and lean proteins foster cellular homeostasis, support metabolic health, and mitigate aging-related damage [61,62].

One of the most extensively studied dietary models is the Mediterranean diet, which has been linked to increased lifespan and reduced chronic disease incidence [63]. This diet, characterized by olive oil [63], abundant fruits and vegetables, whole grains, legumes, nuts, fish, and moderate wine consumption, is associated with a 25% reduction in overall mortality and significantly lower rates of cardiovascular diseases [64]. Its high content of monounsaturated fats, omega-3 fatty acids, and polyphenols provide strong anti-inflammatory and antioxidant benefits. Bioactive compounds like resveratrol and hydroxytyrosol activate sirtuins, proteins involved in cellular repair and longevity, while also reducing pro-inflammatory cytokines such as interleukin-6 (IL-6) and tumor necrosis factor-alpha (TNF-α) [65].

In aging populations, personalized nutritional strategies are essential to address conditions such as diabetes, cardiovascular diseases, osteoporosis, and sarcopenia [66]:

- Diabetes: Low glycemic index diets improve glycemic control and reduce cardiovascular complications. These emphasize whole grains, legumes, nuts, and fresh produce while limiting refined sugars [67].
- Osteoporosis: Adequate calcium, vitamin D, and protein intake maintains bone density and reduces fracture risk, with sources including low-fat dairy, fortified foods, and fatty fish [68].
- Cardiovascular health: Reducing saturated fats and sodium while increasing monounsaturated and polyunsaturated fats supports vascular integrity and reduces oxidative stress. Antioxidant-rich foods, such as berries and leafy greens, further enhance these effects [66].
- Sarcopenia: Adequate protein intake (1.2–1.5 g/kg/day) and leucine-rich foods like poultry and legumes supports muscle protein synthesis and counteracts muscle loss, especially when protein intake is evenly distributed across meals [69].

Micronutrient adequacy is also pivotal in healthy aging. Antioxidants such as vitamins C and E, selenium, and zinc protect cells from oxidative damage. Vitamin D supports bone health, and immune function, while B vitamins (B6, B12, folate) contribute to homocysteine metabolism, reducing cardiovascular and cognitive risks. Magnesium is essential for energy metabolism and DNA repair, while iron and copper are critical for red blood cell production and mitochondrial function [70,71].

Caloric restriction (CR) and intermittent fasting (IF) have gained attention as strategies to delay aging. CR, defined as a reduction in caloric intake without compromising essential nutrients, extends lifespan by reducing oxidative damage, enhancing mitochondrial efficiency, and activating longevity-related pathways such as AMP-activated protein kinase (AMPK) and mammalian target of rapamycin (mTOR). IF promotes autophagy, clearing damaged organelles and proteins to prevent cellular dysfunction [66,72]. Both approaches must be carefully tailored for older adults to balance energy needs and nutrient adequacy.

**Figure 3:**
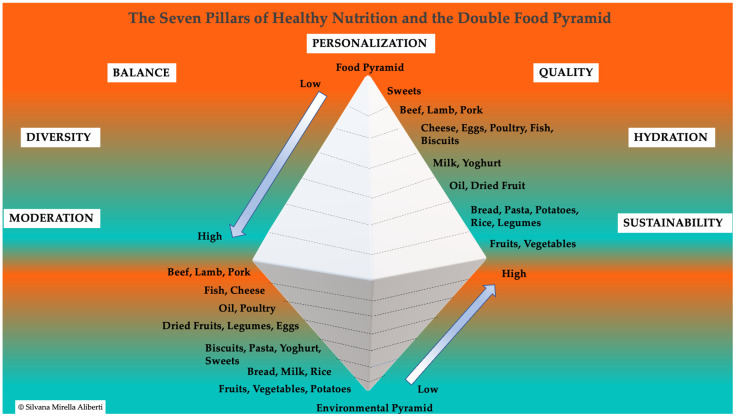
The Seven Pillars of Healthy Nutrition and the Double Food Pyramid: A Holistic Framework Integrating Diet, Health, and Sustainability. Note: This framework was conceptualized by the authors based on an extensive synthesis of scientific research on nutrition, aging, and sustainability. It aims to align health promotion with environmental priorities, offering a comprehensive approach to dietary choices. By integrating of the Seven Pillars with the Double Food Pyramid, this model highlights the dual benefits of dietary patterns that simultaneously support both individual health and environmental sustainability.

To provide a holistic approach to nutrition and aging, the Seven Pillars of Healthy Nutrition [73] were integrated with the Double Food Pyramid [74], creating a comprehensive framework that aligns health promotion with environmental sustainability (Figure 3). This integration highlights:

○ Balance [75]: Achieving optimal macronutrient proportions to support metabolic health.
○ Diversity [76]: Incorporating a wide variety of foods to ensure comprehensive micronutrient intake.
○ Moderation [77]: Encouraging portion control to prevent overnutrition and undernutrition.
○ Quality [78,79]: Prioritizing minimally processed, nutrient-dense foods to maximize health benefits.
○ Hydration [80]: Ensuring adequate water intake to maintain cellular function and overall well-being.
○ Sustainability [75,81]: Making dietary choices that minimize environmental impact while promoting health.
○ Personalization [82,83]: Adapting dietary strategies to individual health status, age, lifestyle, and cultural preferences.

The Double Food Pyramid visually underscores the synergy between individual health and sustainability, emphasizing plant-based foods while de-emphasizing red and processed meats due to their health risks and environmental impact. Foods such as legumes, nuts, and seasonal fruits align with both frameworks by reducing inflammation, supporting metabolic health, and minimizing ecological footprints (Figure 3).

In conclusion, a nutrient-dense and sustainable diet is essential for longevity and resilience against aging-related challenges. Nutritional strategies aligned with models like the Mediterranean diet, Seven Pillars of Healthy Nutrition, and the Double Food Pyramid offer an integrated approach to fostering both individual health and planetary health.

### 3.3. Physical Activity and Functional Longevity

Physical activity is a key determinant of healthy aging, mitigating physiological decline, reducing the risk of chronic diseases, and preserving functional independence [84,85]. Aging is associated with muscle loss, increased body fat, reduced bone density, and cardiovascular and cognitive decline, all of which heighten frailty and fall risk [86,87]. Regular exercise mitigates these effects by improving cardiovascular function, maintaining muscle mass, and enhancing bone density. Aerobic activities (e.g., walking, swimming, cycling) promote cardiovascular efficiency, while strength training helps prevent sarcopenia and osteoporosis [88,89,90].

Beyond physical benefits, exercise plays a crucial role in mental well-being, reducing symptoms of depression, anxiety, and cognitive decline. It promotes neurotransmitter release (serotonin, dopamine), enhancing mood, sleep quality, and psychological resilience [91,92]. Additionally, participation in physical activities fosters social interaction, reducing isolation and strengthening emotional support networks, which are crucial for older adults [93].

From a preventive perspective, regular movement also improves metabolic health by enhancing insulin sensitivity, and lipid profiles, lowering the risk of type 2 diabetes and cardiovascular diseases. Weight-bearing and resistance exercises specifically protect against osteoporosis and joint degeneration [86,94].

Recent research also highlights the impact of physical activity in managing complex health conditions. A study by Aliberti et al. [90] found that engaging in at least six hours of physical activity weekly provides significant benefits for individuals with rare diseases, improving quality of life, social interaction, and weight control. These findings reinforce the need for tailored physical activity recommendations, particularly for individuals with chronic conditions or mobility limitations [90].

The WHO recommends at least 150 min of moderate-intensity aerobic activity or 75 min of vigorous-intensity activity weekly, complemented by strength training on two or more days. For those with mobility impairments, even low-intensity activities such as walking or seated exercises yield substantial benefits [95].

In conclusion, integrating regular physical activity into daily life promotes longevity, preserves cognitive and functional abilities, and enhances overall well-being. As global populations age, ensuring accessibility to exercise programs remains essential for maintaining independence and reducing the burden of age-related diseases (Figure 1).

### 3.4. Microbiome Diversity and Local Epidemiology

The human microbiome, a vast ecosystem of microorganisms residing in the gut, skin, respiratory tract, and mucosal surfaces, plays a fundamental role in health maintenance and the aging process [96]. It regulates nutrient metabolism, immune responses, and cognitive function while providing defense against [97]. Dysbiosis—an imbalance in microbial composition—has been linked to age-related conditions such as cardiovascular disease, neurodegeneration, diabetes, and systemic inflammation, emphasizing the microbiome’s role in longevity and disease prevention [98].

Aging is typically associated with reduced microbiome diversity, characterized by a decline in beneficial bacteria (e.g., Bifidobacterium and Lactobacillus) and an increase in pro-inflammatory species (Firmicutes and Proteobacteria). This shift contributes to “inflammaging”—chronic low-grade inflammation that accelerates atherosclerosis, insulin resistance, cognitive decline. Gut dysbiosis also weakens intestinal barrier integrity, allowing endotoxins like lipopolysaccharides (LPS) to enter the bloodstream and exacerbate systemic inflammation [99].

Diet is a primary factor shaping microbiome composition, with direct implications for health and longevity. Fiber-rich, plant-based diets support microbial diversity and encourage the production of short-chain fatty acids (SCFAs), which enhance gut barrier function and modulate immune responses. Fermented foods such as yogurt, kefir, and kimchi introduce beneficial microbes, further promoting gut health. Conversely, Western-style diets high in processed foods, refined sugars, and unhealthy fats foster dysbiosis, increasing inflammation and metabolic dysfunction [100].

The Mediterranean diet, rich in fiber, polyphenols, and monounsaturated fats, exemplifies a microbiome-friendly dietary pattern. It promotes beneficial bacterial growth while reducing inflammation and metabolic disorders. Similarly, traditional diets such as the Okinawa diet—high in vegetables, soy, and fish—are associated with diverse gut microbiota and reduced chronic disease risk. In contrast, urbanized diets, antibiotic overuse, and environmental pollution contribute to declining microbial diversity and the rise of metabolic and autoimmune disorders [7,101,102,103,104,105,106,107].

The integration of microbiome research into public health strategies presents opportunities to mitigate aging-related diseases. Personalized nutrition, emphasizing fiber-rich and probiotic foods, can enhance microbiome resilience, while advanced therapeutic approaches like fecal microbiota transplants (FMT) and microbiome-targeted therapies hold promise for conditions such as metabolic syndrome, and neurodegeneration.

In conclusion, microbiome diversity is a key determinant of healthy aging, influenced by diet, geography, and environmental exposures. Traditional dietary patterns rich in fiber and fermented foods support microbial balance, reduce inflammation, and promote longevity. Integrating microbiome science into public health initiatives offers a pathway to improved aging outcomes and enhanced quality of life (Figure 1).

### 3.5. Social Support and Psychological Resilience

Social support and psychological resilience are essential for maintaining health, well-being, and longevity in aging populations. Strong social networks—comprising family, friends, and community connection—provide emotional, instrumental, and informational support, helping older adults navigate age-related challenges [6]. Psychological resilience, the capacity to adapt and recover from adversity, plays an equally vital role in maintaining mental and physical health. Together, these factors mitigate stress, reduce the impact of chronic diseases, and promote overall quality of life [108].

Robust social connections have been consistently associated with better health outcomes, including reduced risks of cardiovascular disease, improved immune function, and lower mortality rates [7,109]. House et al. [110] demonstrated that individuals with strong social ties have a significantly lower risk of premature death compared to those who are socially isolated. Social engagement also benefits cognitive health by stimulating mental activity, reducing stress, and fostering emotional stability. Conversely, social isolation and negative interactions contribute to psychological distress, increasing the risk of depression and anxiety [111,112].

Research by Aliberti et al. [6] highlights the evolving role of social support in aging, particularly among nonagenarians and centenarians. While younger older adults often experience psychosomatic issues like sadness, health-related worries, and insomnia, positive social interactions significantly psychological well-being. Support from family members and professional caregivers improves emotional stability and mental health, while participation in cultural programs or honorary achievements fosters a sense of purpose. These findings underscore the shifting dynamics of caregiving, with professional support increasingly supplementing traditional family roles [6].

Psychological resilience further contributes to successful aging by reducing distress and promoting adaptive coping strategies. Resilient individuals experience lower rates of depression, anxiety, and cognitive decline, maintaining a sense of purpose and engagement in life. Key resilience factors include emotional regulation, optimism, and proactive problem-solving, which encourage health-enhancing behavior such as regular physical activity and balanced nutrition [113,114].

Social support reinforces resilience by providing emotional reinforcement during times of stress and strengthening a sense of belonging [6,7]. Cohen and Wills [115] demonstrated that social support buffers the negative effects of chronic stress, while involvement in social groups—such as religious communities, hobby clubs, or volunteer organizations—enhances coping abilities and psychological well-being.

The combination of strong social networks and resilience is essential for longevity. Older adults with close social ties are more likely to engage in health-promoting behaviors, adhere to medical treatments, and experience lower levels of frailty and cognitive decline. Holt-Lunstad et al. [116] found that social isolation poses health risks comparable to smoking and obesity, reinforcing the importance of fostering social connections in aging populations.

In conclusion, social support and psychological resilience are fundamental to healthy aging. By promoting social engagement, emotional support, and resilience-building strategies, societies can improve quality of life, reduce mental health risks, and support longer, healthier lives (Figure 1).

## 4. Risk Factors in Aging and the Transition Between Health and Disease

The process of aging is influenced by a dynamic interaction between genetic predispositions, environmental exposures, and lifestyle choices, which together determine health outcomes and the risk of developing age-related diseases [4,5,117]. While protective behaviors, such as regular physical activity, a balanced diet, and mental well-being, can decelerate aging and reduce the burden of chronic conditions, detrimental environmental factors significantly accelerate cellular degeneration and contribute to increased diseases susceptibility [118]. The modern exposome, encompassing pollutants, synthetic chemicals, and other environmental stressors, play a pivotal role in shaping longevity and health trajectories [119].

Among the most significant environmental risks, exposure to synthetic chemicals and pollutants is particularly concerning. In developed nations, individuals encounter between 80,000 and 100,000 chemical substances in daily life, with certain occupational environments exceeding 200,000 compounds. Many of these xenobiotics interfere with essential physiological processes, promoting premature cellular aging and increasing the prevalence of neurodegenerative disorders, cardiovascular diseases, and cancers [120]. Research in environmental epidemiology has provided robust links between pollution—especially airborne particulate matter, water contaminants, and occupational chemical exposures—and adverse health outcomes, including heightened mortality [121,122]. The Global Burden of Disease (GBD) project attributes approximately 60% of global deaths to modifiable environmental risks, with pollution alone responsible for an estimated nine million deaths annually [123,124].

Environmental toxins accelerate aging by inducing oxidative stress, chronic inflammation, and cellular dysfunction, which are closely tied to conditions such as Alzheimer’s disease, atherosclerosis, and various malignancies [125,126,127]. Airborne pollutants can cross the blood–brain barrier, including neurotoxicity, neuroinflammation, and neuronal dysfunction, contributing to cognitive decline and increased dementia risk [128,129,130,131,132,133,134,135].

Additionally, fine particulate matter (PM2.5) is associated with heightened risks of cardiovascular disease, osteoporosis, and musculoskeletal frailty [136,137,138]. Recent evidence highlights that exposure to plastics and microplastics further compounds these risks, disrupting endocrine function, contributing to metabolic disorders, and increasing cancer susceptibility [135,139,140].

Aging also involves a gradual transition from health to disease, in which individuals accumulate risk factors over time. Initially, those in good health may develop subclinical risk conditions, such as hypertension, metabolic imbalances, or systemic inflammation, due to prolonged exposure to environmental toxins [126,127,128,129,130,131,132,133,134,135,136,137,138,139], poor diet [141,142], or sedentary behavior [142,143]. These conditions progressively weaken the body’s homeostatic mechanisms, increasing the likelihood of chronic disease development [126,127,128,129,130,131,132,133,134,135,136,137,138,139]. However, timely interventions—including lifestyle modifications, medical treatments, and environmental regulations—can effectively mitigate these risks and even reverse early pathological changes, preventing disease onset [144].

**Figure 4:**
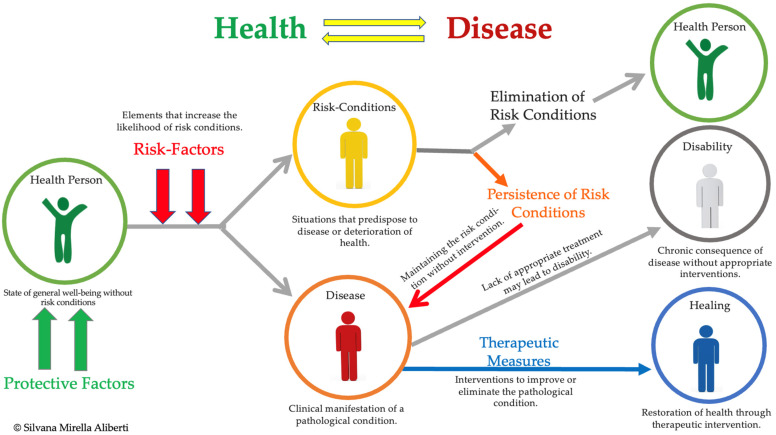
The Health–Disease Continuum: Pathways of Risk Mitigation and Outcomes. A conceptual framework for understanding the interplay of protective factors, risk factors, and therapeutic measures.

The health–disease continuum (Figure 4) illustrates how accumulated risk factors (red) lead to risk conditions (yellow), which, if unaddressed, progress to chronic diseases (red) and, in severe cases, result in disability (gray). Protective factors, including reduced environmental toxin exposure, improved nutrition, and regular physical activity, can slow or halt disease progression (green arrows) and even restore health (blue arrows) [7,87]. Interventions occur at different levels: upstream measures focus on societal and policy-driven efforts to reduce environmental hazards, while downstream interventions target individual health management through clinical treatments and lifestyle adaptations [124].

Older adults are particularly vulnerable to cumulative risk factors. Addressing environmental exposures, promoting early detection of risk conditions, and implementing preventive strategies are critical for enhancing longevity and quality of life. Recognizing the gradual nature of health deterioration allows for targeted interventions that delay or prevent chronic diseases, reinforcing the importance of public health policies in mitigating aging-related risks.

## 5. Longevity in the Blue Zones: Natural Models and Lessons

The concept of Blue Zones [145]—geographically distinct regions where populations exhibit exceptional longevity [146,147]—has attracted significant scientific interest for its potential to uncover pathways to healthy aging. Despite their diverse cultural and environmental contexts, Blue Zones share common dietary, lifestyle, and social factors that contribute to the remarkable health and longevity of their residents [7,148,149,150,151,152]. These zones, which include Okinawa (Japan), Sardinia (Italy), Ikaria (Greece), Nicoya (Costa Rica), Loma Linda (USA), and Martinique (France), offer a unique opportunity to study the interplay between environment, culture, and biology in shaping the aging process (for more comprehensive details, see our previous articles [7]).

Okinawa: A Model of Balance and Purpose

Okinawa is distinguished by its noteworthy prevalence of centenarians, a phenomenon attributable to a lifestyle that emphasizes balance and purpose. This assertion is further substantiated by comprehensive annual demographic data, which reveal an aging tendency ratio of 237.73 in 2023 [153,154,155] (Table 1). The Okinawans’ diet, characterized by a substantial consumption of vegetables, legumes, and sweet potatoes, is inherently plant-based and low in calories [7,156,157,158,159,160,161]. The cultural practice of “Hara Hachi Bu”, which encourages individuals to eat until they are 80% full, is a strategy that aims to prevent overconsumption and promote metabolic health [159]. The integration of physical activity into daily life, such as gardening and walking, is a hallmark of the Okinawan lifestyle. Additionally, the concept of “Moai”, which refers to tight-knit social support networks, plays a pivotal role in fostering emotional well-being. The concept of “Ikigai” (a reason for living) further enhances psychological resilience and purpose, contributing to their longevity [7,159] (Table 2).

Sardinia: A Region of Exceptional Longevity

Sardinia, particularly the Barbagia region, is distinctive among Blue Zones for its exceptional longevity patterns, supported by substantial demographic records [162]. The aging tendency ratio of 268.37 in 2024 [163,164] (Table 1), reflects a significantly aged population, indicative of demographic conditions that sustain longevity. Although the aging tendency ratio does not directly measure the prevalence of centenarians, it provides insight into the overall demographic structure associated with increased life expectancy. Comparisons with non-Blue Zone regions (e.g., Frankfurt am Main, which had an aging tendency ratio of 158.25 in 2023) suggest that Sardinia maintains a uniquely high proportion of older adults, consistent with its recognized longevity patterns.

Sardinia provides a valuable model for the study of longevity. The Sardinian diet is characterized by the consumption of whole grains, legumes, and extra virgin olive oil, with moderate consumption of red wine [146,165,166]. Notably, physical activity remains a central aspect of life, with many residents engaged in farming and shepherding into old age. The region’s strong community and family ties have been shown to promote social cohesion and mental well-being [167,168] (Table 2). The synergistic effect of these elements, in conjunction with genetic predispositions [166], establishes a comprehensive framework for healthy aging.

Ikaria: Stress-Free Longevity

The Greek island of Ikaria was observed to have a remarkably high life expectancy, with a significant proportion of its population living well into their ninth decade and beyond. However, the paucity of recent demographic data, last recorded in 2011, poses challenges in fully evaluating the island’s potential [149,169] (Table 1). The Ikarian diet is characterized by a substantial consumption of vegetables, legumes, and whole grains, complemented by a moderate intake of fish and wine [7,170,171]. The island’s lifestyle, characterized by a focus on physical activity, such as farming and walking, and a relaxed pace of life, contributes to a reduced-stress environment and promotes mental well-being [159]. The practice of napping and the island’s robust social networks further enhance the health and longevity of its residents (Table 2).

Nicoya: Purpose and Natural Resources

Nicoya, Costa Rica, is noteworthy for its notable population of centenarians. Despite the paucity of data regarding nonagenarians and centenarians, Nicoya’s aging tendency ratio was 117.03 in 2020 [172,173] (Table 1). The diet of Nicoyans is characterized by a nutritional pattern centered on corn, beans, squash, and tropical fruits, supplemented by calcium-rich hard water, which contributes to optimal bone health [159,174,175,176,177]. The community’s strong family bonds and a distinct sense of purpose, known as “plan de vida”, play a pivotal role in their cultural identity [7,159]. Physical activity, predominantly through farming and walking, is a daily norm [178] (Table 2). However, challenges persist, including incomplete data on nonagenarians and centenarians, underscoring the necessity for systematic updates to enhance our understanding of longevity in this region [172,173,179] (Table 1).

Loma Linda: Faith-Based Longevity

In Loma Linda, California, the Seventh-Day Adventist serves as a paradigm of faith-based healthful living [7]. While earlier demographic studies are available, the absence of precise official registry data for nonagenarians and centenarians curtails the capacity for exhaustive analysis. Their aging tendency ratio was recorded at 174.21 in 2023 [180] (Table 1). Their diet, predominantly plant-based with a high consumption of legumes, nuts, and whole grains, is complemented by abstinence from alcohol and tobacco [181]. Regular physical activity and spiritual practices, including weekly Sabbath observance, contribute to mental and physical well-being. The presence of robust social networks within the community further contributes to their enhanced quality of life and longevity [182,183,184,185,186] (Table 2).

Martinique: A New Addition

Martinique, as a Blue Zone in 2022, offers insights into longevity in a Caribbean context, with an aging tendency ratio of 239.75 in 2023 [187,188] (Table 1). The diet includes tropical fruits, vegetables, beans, and fish, emphasizing local and minimally processed foods. Social cohesion and family bonds are central to Martinican culture, while traditional forms of dance and walking integrate physical activity into daily routines [189] (Table 2). Despite the availability of official records for aging tendencies and nonagenarians, confirming centenarian data required manual efforts due to the lack of a centralized population register [190,191] (Table 1). Michel Poulain’s identification of Martinique as a Blue Zone underscores its potential for further demographic and longitudinal studies to explore the mechanisms underlying its longevity patterns.

### Cilento as an Emerging Model

Cilento, located in the Campania region of southern Italy, presents a distinctive ecological and cultural milieu that closely mirrors the characteristics of established Blue Zones [7]. Partly recognized as a UNESCO World Heritage Site, the region spans approximately 490,000 hectares and 102 municipalities, encompassing a diverse landscape of coastal, hilly, and mountainous areas [4] enriched by karst phenomena [192,193] and scattered springs [194]. This natural diversity has fostered a deep interaction between environmental exposures and human adaptation, shaping longevity-related biological mechanisms [4].

Cilento’s longevity is particularly evident in its central municipalities, especially in the hilly areas at altitudes between 440 and 600 m. The region’s transitional climate, characterized by mild temperatures averaging 20 °C (68 °F), moderately dry summers, and wet winters, has been associated with reduced cardiovascular risk and enhanced general health benefits [4]. Beyond climate, environmental exposures in Cilento, such as biodiversity, water quality, and air purity, contribute to long-term health by modulating inflammatory responses and metabolic efficiency. These elements align with the exposome framework, which evaluates cumulative lifetime exposures affecting aging trajectories.

A key factor in Cilento’s longevity is its adherence to the Mediterranean diet, originally defined by Ancel Keys during his studies in the region in the 1960s [195]. This diet, rich in extra virgin olive oil (EVO), wild greens, legumes, and locally sourced products, supports cardiovascular and metabolic health through bioactive compounds with anti-inflammatory properties [7]. A major advancement of this study compared to previous research [7] (Aliberti et al., 2024) is the incorporation of the microbiome as a fundamental determinant of longevity. The high dietary diversity in Cilento promotes a balanced gut microbiota, which plays a crucial role in immune function, metabolic regulation, and age-related diseases [196,197].

**Table 1:** Aging tendency ratio and demographic data availability of blue zones and Cilento.

Region	Availability of Data	Aging Tendency Ratio [4]	Specific Issues or Notes	* Evaluation (Score)
Okinawa	Comprehensive annual demographic data, including centenarians [153].	237.73 in 2023	Robust demographic datasets with consistent updates since 1975. However, earlier records are less comprehensive, creating potential gaps in historical analyses. This limitation is partially mitigated by high-quality recent data [154,155].	5/5
Sardinia	Consistent and robust records of aging demographics [163,164], with a notable focus on male centenarians [162].	268.37 in 2024	Extensive demographic records, particularly on centenarians, supported by national [163] and local [164] data sources. No major limitations identified, as available data provide a strong foundation for research [145,162].	5/5
Ikaria	Limited availability of annual demographic data. The most recent comprehensive dataset originates from the 2011 census [149,169]	Last recorded 252.82 in 2011 [169]	Limited availability of demographic records and absence of detailed, age-stratified data. Reliance on fragmented sources and local observations hinders trend analysis for nonagenarians and centenarians, limiting longitudinal research potential.	2/5
Nicoya	Incomplete data on age-stratified demographics; lacks regular updates for nonagenarians and centenarians [172].	117.03 in 2020	Archivial data and local studies exist but lack systematic updates and detailed age-stratified demographic tracking. This limitation hinders comprehensive analysis and long-term research comparison.	3/5
Loma Linda	Early demographic studies available; no precise official registry data for nonagenarians or centenarians [180].	174.21 in 2023	Demographic insights rely on community-specific studies and historical records. However, the absence of official population registry for nonagenarians and centenarians results in notable gaps in statistical accurancy.	4/5
Martinique	Reliable records for aging tendencies and nonagenarians are available. However, identifying centenarians remains challenging due to incomplete and fragmented population registers [187,188].	239.75 in 2023	Reliable data on aging tendency and nonagenarians. However, the absence of a comprehensive population registry complicates accurate centenarian identification. Michel Poulain et al. [190] who recently recognized Martinique as a Blue Zone, highlighted the considerable manual effort required for record validation. The region holds strong research interest, but systematic data collection needs improvement.	4/5
Cilento	Comprehensive and recent demographic data with detailed annual updates on aging tendencies and centenarians [163].	251.19 in 2024	Comprehensive and regularly updated demographic data on aging and centenarians. Supported by official records and extensive studies [4,5,7], it serves as a model for demographic research in longevity hotspots. No significant limitations identified.	5/5

Notes: Aging tendency—the ratio of 65+ population to the total population—is used to reflect the total local old population and the aging trend (see our previous study [4] for more information). * Evaluation Criteria—The evaluation score was assigned based on the quality and completeness of available demographic data. Factors considered included the presence of formal registries, the stratification of data by advanced age groups, and the frequency of updates. Official records, demographic studies, and expert analyses (e.g., Poulain’s research) were used to assess data reliability and limitations. The scoring was conducted independently by two researchers, with final agreement reached through consensus. The availability of annual demographic data is critical for maintaining the scientific validity and recognition of these regions as unique models of longevity.

Building upon prior comparative studies, this research also introduces a novel evaluation score based on the exposome model, systematically assessing the impact of environmental, nutritional, physical activity, microbiome, and psychosocial factors on longevity. Cilento’s aging tendency ratio of 251.19 (2024) [163] underscores its relevance as a model for healthy aging. Comparative analyses, as presented in Table 1 and Table 2, highlight common longevity determinants across different regions; however, Cilento presents unique interactions between environmental exposures, biological adaptations, and microbiome composition, distinguishing it from the traditionally recognized Blue Zones.

The following section explores parallels between Cilento and other longevity hotspots (Okinawa, Sardinia, Ikaria, Nicoya, Loma Linda, and Martinique), emphasizing shared protective factors and region-specific traits.

- Cilento and Okinawa

Cilento and Okinawa share core longevity determinants shaped by environmental exposures and lifestyle practices, though their dietary and cultural components differ. The Mediterranean diet in Cilento emphasizes extra virgin olive oil (EVO), figs, and locally cultivated vegetables such as chicory and borage [7], while Okinawa’s dietary model is rich in sweet potatoes, tofu, and miso [155,156]. Both diets provide high concentrations of polyphenols, flavonoids, and omega-3 fatty acids, contributing to cardiovascular protection, oxidative stress reduction, and improved metabolic efficiency [104,105]. These nutritional components actively influence gut microbiome diversity, a key mediator in the aging process [7,101].

Physical activity patterns in both regions integrate naturally into daily life, reinforcing the role of environmentally driven movement in longevity. In Cilento, agricultural labor and pastoral traditions maintain musculoskeletal function and metabolic homeostasis, paralleling Okinawa’s older engagement in gardening and traditional crafts. Furthermore, psychosocial factors play a crucial role in resilience and longevity: Okinawa’s concept of ikigai (sense of purpose) finds its counterpart in Cilento’s deep-rooted intergenerational ties, where family networks and profound connection to the land provide stability, emotional well-being, and social engagement. Okinawa’s moai (lifelong social support groups) mirror the strong communal structures in Cilento, where shared meals, local festivals, and rural solidarity networks foster a sense of belonging. These social frameworks contribute to stress modulation and enhanced immune function, aligning with exposome-driven models of psychosocial resilience and healthspan extension [7].

- Cilento and Sardinia

The Mediterranean diet forms the backbone of both Sardinia and Cilento, with shared staples such as olive oil, legumes, and whole grains. Sardinia’s traditional dishes, like sourdough bread and minestrone, align with Cilento’s nutrient-dense meals such as *strinta* soup [7]. These diets, rich in polyphenols and anti-inflammatory compounds, contribute to cardiovascular and metabolic health. Additionally, Cilento offers a unique opportunity to evaluate the role of the gut microbiome in longevity, as its dietary diversity fosters a beneficial microbial composition, a factor not previously explored in comparative analyses.

From an environmental perspective, both regions share a mild Mediterranean climate, which facilitates outdoor physical activities and agricultural work. The rural lifestyle in Sardinia and Cilento promotes natural movement, particularly in farming and animal husbandry, reinforcing the link between physical activity and longevity. Moreover, both regions exhibit strong intergenerational relationships that support mental and emotional health. Sardinia’s social structure, characterized by close-knit family networks, mirrors Cilento’s communal lifestyle, where shared meals and collective traditions reinforce social bonds and psychological resilience [7]. These factors, when analyzed through the exposome framework, reveal distinctive interactions between environmental exposures, lifestyle factors, and biological adaptations that contribute to longevity in Cilento.

- Cilento and Ikaria

Cilento and Ikaria share a deep cultural connection to the Mediterranean diet. In Ikaria, olive oil, legumes, and herbal teas dominate, mirroring Cilento’s focus on olive oil, figs, and diverse vegetables. Both regions emphasize local and seasonal foods, which are rich in antioxidants and contribute to longevity [7]. Notably, the inclusion of the gut microbiome in this analysis provides additional insight into how dietary patterns in these regions influence microbial diversity, immune function, and metabolic health, an aspect that enhances this study compared to previous research.

The natural environment in both Ikaria and Cilento fosters physical well-being. The hilly terrains encourage regular movement, while the mild Mediterranean climate reduces environmental stressors that could accelerate aging. Social cohesion is another significant commonality; Ikaria is known for its communal gatherings and shared meals, which are reminiscent of Cilento’s intergenerational family traditions and a strong sense of community. Additionally, both regions value a slower pace of life, reducing stress and supporting mental well-being [7]. By applying the exposome framework, this study highlights how the interplay between environmental exposures, diet, and psychosocial resilience creates a distinctive longevity model in Cilento, setting it apart from other Blue Zones.

- Cilento and Nicoya

Cilento and Nicoya share a dietary emphasis on plant-based foods, though the specifics differ due to cultural and geographic factors. In Nicoya, staples include beans, corn, and squash, while Cilento’s diet features olive oil, figs, and vegetables like chicory and borage. However, both dietary patterns promote gut microbiome diversity through high fiber intake and polyphenol-rich foods, which contribute to reduced inflammation and improved metabolic function.

From a lifestyle perspective, both regions integrate physical activity into daily routines. In Nicoya, agricultural work and forestry promote movement, while in Cilento, longevity is linked to farming and pastoral activities. Social structure also plays a fundamental role in both regions: Nicoya’s strong intergenerational ties and sense of *plan de vida* reflect the deeply rooted familial and communal relationships in Cilento. These social dimensions, coupled with a nutrient-rich diet and daily physical activity, contribute to resilience, psychosocial well-being, and longevity [7].

- Cilento and Loma Linda

Cilento and Loma Linda exhibit similarities in their emphasis on healthy diets, though their approaches differ. Loma Linda’s Adventist community follows a whole-food, plant-based diet, which excludes meat and focuses on nuts, fruits, and vegetables. Cilento’s Mediterranean diet, while including moderate amounts of animal products, shares a common emphasis on antioxidant-rich foods such as olive oil, legumes, and fresh produce. Both dietary patterns have been associated with beneficial gut microbiome profiles, characterized by an increased presence of short-chain fatty acid-producing bacteria, which play a crucial role in reducing systemic inflammation and supporting metabolic health.

Both regions promote active lifestyles, though Loma Linda emphasizes walking and volunteering, while Cilento integrates physical activity through agricultural and pastoral work. Additionally, social connections are vital in both regions in Loma Linda, community support is enhanced by shared religious practices, while in Cilento, strong family and communal relationships provide emotional resilience [7]. These social structures, combined with diet that nurtures a healthy gut microbiota, may offer protective effects against age-related diseases.

- Cilento and Martinique

Cilento and Martinique share several similarities in their dietary and environmental factors. Martinique’s traditional diet is rich in fresh fruits, vegetables, and tubers, paralleling Cilento’s emphasis on figs, olive oil, and seasonal vegetables. Both regions prioritize minimally processed, nutrient-dense foods that provide antioxidants and support metabolic health [198]. The high dietary fiber intake in both regions likely fosters a beneficial gut microbiota composition, enhancing immune function and reducing the risk of chronic inflammation-related conditions.

The climate in both regions plays a significant role in fostering longevity. Cilento’s mild Mediterranean climate and Martinique’s tropical climate encourage outdoor activities and physical labor, such as farming and gardening [7,199,200]. Regular exposure to nature and physical activity are known to influence gut microbiome diversity, with potential benefits for overall health and longevity.

Socially, Martinique’s strong family structures and sense of community echo Cilento’s intergenerational bonds and communal living [7,201,202]. Both regions emphasize the importance of traditional knowledge and cultural heritage, which support mental well-being and provide a sense of purpose. The combination of a nutrient-dense diet that supports a balanced gut microbiome, regular physical activity, and strong social support networks contributes to a holistic model of healthy aging, reinforcing the role of environmental and lifestyle factors in shaping longevity outcomes.

**Table 2:** Assessment of key protective factors for longevity: a comparative analysis of Blue Zones and Cilento.

Factors	Blue Zones (Okinawa, Sardinia, Nicoya, Loma Linda, Ikaria, Martinique)	Cilento	Rationale	* Evaluation (Blue Zones)	* Evaluation (Cilento)
Natural Environment (air, biodiversity)	Altitude: ~400–600 m; Climate mean temperature ~17–25 °C; Clean air, biodiversity [7,145,147].	Altitude: 400–600 m; Mediterranean climate ~20 °C; Clean air, biodiversity [4,7].	Both regions benefit from moderate altitude, mild climate, and high biodiversity, which promote physical and mental health through reduced stress and improved quality of life.	5	5
Diet (Sustainable Nutrition)	Plant-based diet rich in legumes, whole grains, vegetables, and olive oil [7,146,151,157,158,159,160,165,166,171,175,181,189]	Mediterranean diet rich in olive oil, legumes, vegetables, whole grains, red wine and honey [7,196,197]	Both diets are nutrient-dense and support cardiovascular health. Cilento’s diet aligns closely with Blue Zones’ principles, emphasizing plant-based foods and healthy fats.	5	5
Physical Activity (Natural Movement)	Daily activities like walking, gardening, light physical work [7,151,157,159,167,184]	Farming, daily walking, and moderate physical work [7]	Natural movement is embedded in daily routines in both settings, reducing sedentary behavior and promoting longevity through improved cardiovascular and muscular-skeletal health.	5	5
Microbiome (Gut Health)	Diets rich in fiber and fermented foods promote a healthy microbiome [7,160,197]	Fiber-rich Mediterranean diet with fermented foods [7,196,197]	Both regions prioritize diets that support gut health, reduce inflammation, and increase systemic resilience—critical factors in aging and longevity.	5	5
Social Support (Family and Community Networks)	Strong family ties, close-knit communities [7,159,167,184,201]	Strong family ties, social cohesion [6,7]	Community and family connections are integral to psychological well-being and stress reduction, fostering a sense of belonging that is crucial for healthy aging.	5	5
Psychological Resilience (Stress Management, Sense of Purpose)	Slow lifestyle, strong sense of purpose, spiritual practices [7,149,159,201]	Slower lifestyle, strong sense of purpose [6,7]	Stress management is achieved through cultural and spiritual practices in both regions, supporting mental health and fostering resilience against aging-related challenges.	5	5

* The scoring system evaluates key protective factors in longevity regions using a 5-point scale (1 = minimal evidence, 5 = strong evidence). An expert panel of eight members—including two researchers (Silvana Mirella Aliberti and Mario Capunzo) and six specialists from diverse fields—applied a modified Delphi methodology with iterative rounds to refine scoring and reach consensus. Kendall’s coefficient of concordance (W = 0.87, *p* < 0.001), confirmed a high level of agreement among experts. Further methodological details are provided in Method section.

Despite cultural and geographical differences, the highlighted factors exhibit remarkable consistency between Blue Zones and Cilento. This suggests the existence of universal principles for promoting longevity that transcend regional contexts. Public health strategies inspired by these regions could leverage the exposome framework to design interventions that promote plant-based diets rich in microbiome-supporting nutrients, urban planning that encourages natural movement, and initiatives to reinforce social cohesion and intergenerational connections.

By integrating insights from these longevity hotspots, policymakers and community leaders can foster environments that not only support aging populations but also promote long-term health resilience. The case of Cilento, with its unique combination of protective factors, offers a valuable model for developing evidence-based strategies to enhance well-being and longevity on a broader scale.

## 6. Implications for Preventive Medicine

The synthesis of findings on the exposome’s role in aging, combined with the longevity patterns observed in the Blue Zones and Cilento, provides a compelling framework for reshaping preventive medicine. These insights highlight the need for a multi-level approach, spanning population-wide policies, clinical interventions, and community-based programs, to promote healthy aging.

At population level, environmental factors play a fundamental role in shaping health outcomes. The clean air, biodiversity, and favorable climates of long-lived regions underscore the need for policies that enhance access to green spaces, improve air quality, and support sustainable ecosystems [33,35]. Urban planning should integrate walkable spaces, community gardens, and air quality monitoring [203], while conservation efforts should prioritize biodiversity to sustain nutrient-rich local food supplies [204].

In clinical practice, dietary interventions represent a cornerstone of preventive strategies. The plant-based, nutrient-dense diets observed in long-lived populations, rich in legumes, whole grains, vegetables, and healthy fats, provide a model for nutrition-based health promotion [60,61,62,63]. Personalized nutrition strategies, incorporating microbiome-friendly dietary practices such as high-fiber intake and fermented foods, could enhance gut health, reduce inflammation, and improve systemic resilience against age-related diseases [100,101,102,103].

Physical activity is another key determinant of longevity. The lifestyles of Blue Zone residents and Cilento centenarians emphasize natural movement, including farming, gardening, and walking. Beyond aerobic exercise, emerging evidence highlights the importance of strength training in preserving muscle mass, maintaining metabolic health, and preventing frailty in aging populations. Integrating resistance exercise into preventive health guidelines—through clinical recommendations, workplace wellness programs, and community fitness initiatives—could significantly enhance functional longevity [84,85].

Social cohesion [6] and psychological resilience [108] also play critical roles in health outcomes. Strong family bonds, community networks, and shared cultural practices provide emotional support and mitigate stress-related health risks [110,112]. Preventive strategies should include initiatives to combat social isolation, foster intergenerational engagement, and promote purpose-driven activities, particularly for older adults.

Advancements in exposome science and geroscience offer new opportunities to refine these strategies. Wearable technology and biomonitoring tools enable real-time tracking of environmental exposures and physiological aging markers, allowing for early interventions tailored to individual risk profiles [205]. The “geroscience hypothesis”, which suggests that delaying biological aging can postpone the onset of chronic diseases, provides a framework for addressing aging at its root causes, while traditionally focused on molecular and cellular pathways, this approach increasingly recognizes the social exposome—the cumulative impact of social determinants on biological aging—as a critical target for intervention [206]. Addressing factors such as chronic stress and socioeconomic disparities through community programs and public policies could reduce key drivers of accelerated aging, including systemic inflammation and epigenetic alterations.

From a public health perspective, integrating these insights into educational campaigns and healthcare policies could drive widespread adoption of preventive aging strategies. Multisectoral collaborations, between governments, healthcare providers, and local communities, are essential to translating longevity research into tangible health benefits. By aligning environmental, nutritional, and lifestyle-based interventions with advances in exposome science, preventive medicine can foster healthier aging trajectories at both individual and social levels.

## 7. General Conclusions

This mixed-methods analysis highlights the complex interplay of protective and risk factors in aging and longevity, integrating a systematic review of the exposome’s role with case studies on Blue Zones and Cilento. By combining epidemiological insights, geroscience principles, and microbiome research, this study provides a multidimensional perspective on the determinants of healthy aging.

The findings reinforce the cumulative effect of the exposome, including environmental quality, dietary patterns, physical activity, and psychosocial factors, in shaping longevity outcomes. The comparative analysis of long-lived populations underscores the universal principles of longevity, despite cultural and geographical differences, while also highlighting the distinctive characteristics of Cilento as an emerging natural model.

A key contribution of this study is the integration of microbiome research into the exposome framework. The gut microbiome emerges as a crucial mediator linking diet, environment, and aging-related resilience. Future research should further investigate the dynamic interactions between microbiota composition, dietary diversity, and systemic inflammation to refine targeted interventions for longevity.

Moreover, the application of geroscience to population health suggests that delaying the biological mechanisms of aging could reduce the burden of age-related diseases. Bridging geroscience with public health strategies—through early-life interventions, community-based programs, and personalized lifestyle modifications—represents a promising avenue for extending healthspan at the population level.

Technological advancements in exposome science, such as biomonitoring tools and precision public health strategies, offer new opportunities for assessing environmental risks and tailoring interventions to individual and community needs. These innovations can enable real-time monitoring of biological aging markers, optimizing preventive medicine approaches.

Additionally, our findings emphasize the need to consider multiple demographic indicators when assessing longevity patterns. While the aging tendency ratio serves as a robust measure of population aging trends, centenarian prevalence remains a key parameter for understanding extreme longevity. Future studies should integrate both indicators to provide a more comprehensive assessment of longevity dynamics across different regions.

By integrating case-study insights with systematic exposome analysis, this study provides a roadmap for advancing healthy aging research and policy. Translating these findings into actionable strategies requires interdisciplinary collaboration across epidemiology, biology and public health. Strengthening partnerships between researchers, policymakers, and local communities will be essential to developing sustainable, culturally adaptive longevity-promoting interventions.

Ultimately, harnessing the power of exposome within comprehensive public health frameworks can pave the way for a global paradigm shift in aging, ensuring longer, healthier lives for future generations.

## Data Availability

The datasets are available upon request from the corresponding author via email, subject to privacy and ethical restrictions.
